# Pilot study of gadoxetate disodium-enhanced mri for localized and metastatic prostate cancers

**DOI:** 10.1038/s41598-021-84960-w

**Published:** 2021-03-11

**Authors:** Sarah E. Lochrin, Baris Turkbey, Billel Gasmi, Keith Schmidt, Jonathan D. Strope, Cindy H. Chau, Tristan M. Sissung, Douglas K. Price, Lisa Cordes, Suzana Markolovic, Bradford J. Wood, Peter A. Pinto, Yolanda L. McKinney, Joanna H. Shih, Elliot Levy, Ravi Madan, William Dahut, Peter L. Choyke, Maria Merino, William D. Figg

**Affiliations:** 1 Clinical Pharmacology Program, Center for Cancer Research, National Cancer Institute, Bethesda, MD USA; 2 Molecular Imaging Branch, Center for Cancer Research, National Cancer Institute, Bethesda, MD USA; 3 Translational Surgical Pathology Section, Center for Cancer Research, National Cancer Institute, Bethesda, MD USA; 4 Genitourinary Malignancies Branch, Center for Cancer Research, National Cancer Institute, 9000 Rockville Pike, Building 10, Room 5A03, Bethesda, MD 20892 USA; 5 Center for Interventional Oncology, Radiology and Imaging Sciences, Clinical Center, Center for Cancer Research, National Cancer Institute, Bethesda, MD USA; 6 Urologic Oncology Branch, Center for Cancer Research, National Cancer Institute, Bethesda, MD USA; 7 Biometric Research Program, Division of Cancer Treatment and Diagnosis, National Cancer Institute, Rockville, MD USA; 8 Radiology and Imaging Sciences, Center for Cancer Research, National Cancer Institute, Bethesda, MD USA

**Keywords:** Prostate cancer, Cancer imaging, Tumour biomarkers

## Abstract

OATP1B3 is expressed de novo in primary prostate cancer tissue and to a greater degree in prostate cancer metastases. Gadoxetate disodium is a substrate of OATP1B3, and its uptake has been shown to correlate with OATP1B3 expression in other cancers. We aimed to evaluate use of gadoxetate disodium to image prostate cancer and to track its utility as a biomarker. A single center open-label non-randomized pilot study recruited men with (1) localized, and (2) metastatic castration resistant prostate cancer (mCRPC). Gadoxetate disodium-enhanced MRI was performed at four timepoints post-injection. The Wilcoxon signed rank test was used to compare MRI contrast enhancement ratio (CER) pre-injection and post-injection. OATP1B3 expression was evaluated via immunohistochemistry (IHC) and a pharmacogenomic analysis of OATP1B3, NCTP and OATP1B1 was conducted. The mCRPC subgroup (*n* = 9) demonstrated significant enhancement compared to pre-contrast images at 20-, 40- and 60-min timepoints (*p* < 0.0078). The localized cancer subgroup (*n* = 11) demonstrated earlier enhancement compared to the mCRPC group, but no retention over time (*p* > 0.05). OATP1B3 expression on IHC trended higher contrast enhancement between 20–40 min (*p* ≤ 0.064) and was associated with contrast enhancement at 60 min (*p* = 0.0422). OATP1B1 haplotype, with N130D and V174A substitutions, impacted enhancement at 40–60 min (*p* ≤ 0.038). mCRPC lesions demonstrate enhancement after injection of gadoxetate disodium on MRI and retention over 60 min. As inter-individual variability in OATP1B3 expression and function has both predictive and prognostic significance, gadoxetate disodium has potential as a biomarker in prostate cancer.

## Introduction

Androgen deprivation (ADT) has been shown to decrease serum testosterone by > 90% with a corresponding 75% reduction in intra-tumoral testosterone concentration^1^. Further studies have shown that castration resistant prostate cancer (CRPC) metastases have greater intra-tumoral androgen concentration compared to tumors in eugonadal men^2^. The source of tumor tissue androgens despite ADT has not been fully elucidated but may reflect de novo androgen synthesis, androgen promiscuity and/or androgen scavenging with increased uptake^2^.

OATP1B3 (encoded by *SLCO1B3*) is an uptake transporter and a member of the organic anion transporting polypeptides (OATP) superfamily of transporters. OATP1B3 is expressed on the plasma membrane of hepatocytes and has also been identified in multiple cancer tissues, including colorectal, testicular, pancreatic, breast, lung, endometrial and prostate cancer^3–5^. Its role in bile, steroid and anti-cancer drug transport has been extensively studied to date^3^. OATP1B3 facilitates the diffusion of unconjugated testosterone in several cell types, including prostate cancer^5,6^.

OATP1B3 is expressed de novo in prostate cancer as compared to surrounding normal prostate tissue and is expressed more frequently and to a greater degree in prostate cancer metastases, and therefore potentially plays a role in mediating intra-tumoral androgen concentration^4–8^. OATP1B3-mediated testosterone uptake is greater in cells expressing certain OATP1B3 variants (112S and 233M), and these variants are also associated with poor overall survival from diagnosis, poor progression-free survival on androgen deprivation therapy, biochemical recurrence, and prostate cancer-specific mortality^6,7,9,10^. Tumoral OATP1B3 expression is related to the clinical response of patients with prostate cancer treated with abiraterone and has been shown to determine intracellular concentrations of docetaxel and cabazitaxel in patient-derived xenografts^11,12^.

The use of non-invasive imaging techniques to increase our understanding of prostate cancer development, progression and to aid physicians in risk stratification is a rapidly expanding field^13^. Gadoxetate disodium (Gadoxetic acid, Gd-EOB-DTPA; Eovist) is an FDA-approved gadolinium-based hepatoselective MRI contrast agent that has a proven role in improving the diagnosis and classification of liver lesions^14,15^. Gadoxetate is a substrate for several bile acid uptake transporters; OATP1B3, OATP1B1 (encoded by *SLCO1B1*), and NTCP (encoded by *SLC10A1*)^16,17^. Of these transporters, histopathological OATP1B3 expression demonstrates the strongest correlation with gadoxetate uptake in HCC^17,18^. Frequently occurring polymorphisms in *SLCO1B1* and *SLCO1B3* are of functional relevance in gadoxetate cellular uptake^19,20^.

We hypothesized that gadoxetate disodium-enhanced MRI may provide additional prognostic information and we aimed to evaluate use of gadoxetate disodium to image prostate cancer and to track its utility as a biomarker for non-invasive assessment of the expression of OATP1B3 in prostate tumors.

## Methods

### Patients

Male subjects were recruited and enrolled in a single center open-label non-randomized pilot study between July 2013 and March 2016 (ClinicalTrials.gov identifier—NCT01867424) and were divided into two arms; (1) subjects with localized prostate cancer, and (2) subjects with mCRPC. Medications that were good OATP1B3 substrates or were known to affect its function were held for at least three days prior to MRI. Study patients’ electronic medical records were reviewed, providing demographic information; age, race, BMI, and clinical information; Gleason score, tumor stage and on-study PSA. Informed consents were obtained from all participants prior to trial participation, and the study was approved by the National Cancer Institutional Review Board, and all participants provided written informed consent. All research methods were performed in accordance with relevant guidelines and regulations.

### Imaging technique

Pre-contrast T1 and T2 weighted multi-parametric scans, using a clinical 3T MRI scanner (Philips, USA), with a 32-channel phased array surface coil (In-Vivo Inc), were obtained through the prostate gland, bone or soft tissue metastasis selected as the target lesion. Contrast-enhanced MRI was performed after a rapid bolus injection of gadoxetate disodium (Eovist, Bayer Healthcare Pharmaceuticals, Germany) at a dose of 0.1 ml/kg administered at a rate of 2-ml/second, followed by a 10-ml saline flush (0.9% w/v sodium chloride at 1-ml/second rate). Multi-slice 2D-T1W TSE images with echo and repetition time (TE/TR) of 8.2/312.0 ms were obtained at four timepoints, 10, 20, 40 and 60 min, after injection. In addition to contrast enhanced MRI, axial T2W and diffusion weighted MRI pulse sequences were obtained to locate the target lesions.

### Imaging analysis

The MR imaging was reviewed by a genitourinary radiologist (10 years of experience in prostate and body imaging, BT). The radiologist was blinded to the pathology and demographic data. A region of interest was drawn over the target lesion in the pre-contrast and post-gadoxetate injection MR images and the amount of contrast enhancement was recorded in picture archiving communication system (PACS) (Carestream, Rochester, NY, USA). For the localized disease arm, the lesion with the largest size and highest Gleason score was chosen as the target lesion, whereas for the mCRPC the lesion which is amenable to image guided biopsy was chosen as the target lesion. For each patient, one target lesion was chosen. Contrast enhancement ratios (CER) [(signal intensity after enhancement-signal intensity before enhancement)/signal intensity before enhancement] were calculated by manually drawing region of interests on target lesions on pre- and post- injection T1W MR images and determined at each time point.

### Histopathological analysis

Subjects with localized prostate cancer had image-guided-biopsy-confirmed prostate cancer and sufficient tissue available for OATP1B3 immunohistochemistry (IHC). Subjects with advanced disease had soft tissue or bone lesions underwent percutaneous needle biopsy to obtain tissue for IHC. Formalin-Fixed Paraffin-Embedded (FFPE) tissue specimens were stained with a rabbit polyclonal anti-human OATP1B3 antibody targeting the C-terminal sequence (Antigen sequence SKTCNLDMQDNAAA) (Sigma-Aldrich, St. Louis, MO, USA) as previously described^21^. The staining intensities were evaluated by a pathologist (> 20 years of experience in prostate pathology, MJM) using the following categories: negative, weakly positive (+ 1), moderately positive (+ 2), and strongly positive (+ 3). The pathologist was blinded to the patient demographic and imaging data.

### Genotyping

Genotyping was performed using genomic DNA isolated from stored frozen serum using the QIAmp DNA blood mini-kit (Qiagen, Inc, Valencia, CA). *SLCO1B3* single nucleotide polymorphisms (SNP) 334T>G (rs4149117) and 688 G>A (rs7311358) were genotyped via direct sequencing, as previously published^6^. Additionally, to evaluate the 51 main SNPs in *SLCO1B3*, *SLCO1B1* and *SLC10A1* (NTCP), isolated genomic DNA was genotyped by Pharmacoscan assay (Thermo Fisher Scientific, Waltham, MA, USA), as previously described^22^.

### Statistical analysis

All statistical analyses were performed using GraphPad Prism version 8.4.2, GraphPad Software (San Diego, California USA). Data are expressed as mean  + /− standard deviation (SD). The Wilcoxon signed rank test was used to compare MRI contrast enhancement ratio (CER) pre-injection and post-injection, to evaluate the primary endpoint of uptake and retention of gadoxetate in prostate cancer in each disease stage. For our exploratory analyses, Mann Whitney tests were performed to compare gadoxetate enhancement with stage of disease, Gleason score, histopathologic OATP1B3 expression, and genotype. Spearman correlation assessed the relationship of gadoxetate enhancement with baseline PSA. All p-values are two-tailed and given the exploratory nature of this study a value of *p* < 0.05 was considered to be statistically significant.

## Results

### Patient characteristics

Twenty-four patients were enrolled on study, including 12 subjects with histologically confirmed localized prostate cancer and 12 subjects with mCRPC. Patient characteristics are summarized in Supplementary Table 1*.* The final study population for the primary end point consisted of 20 evaluable patients, who were divided into two arms; 11 subjects with localized prostate cancer and 9 subjects with mCRPC. We excluded patients with nondiagnostic baseline imaging (n = 1) and patients who did not complete the study (n = 3).

### Enhancement pattern in prostate cancers

**Figure 1:**
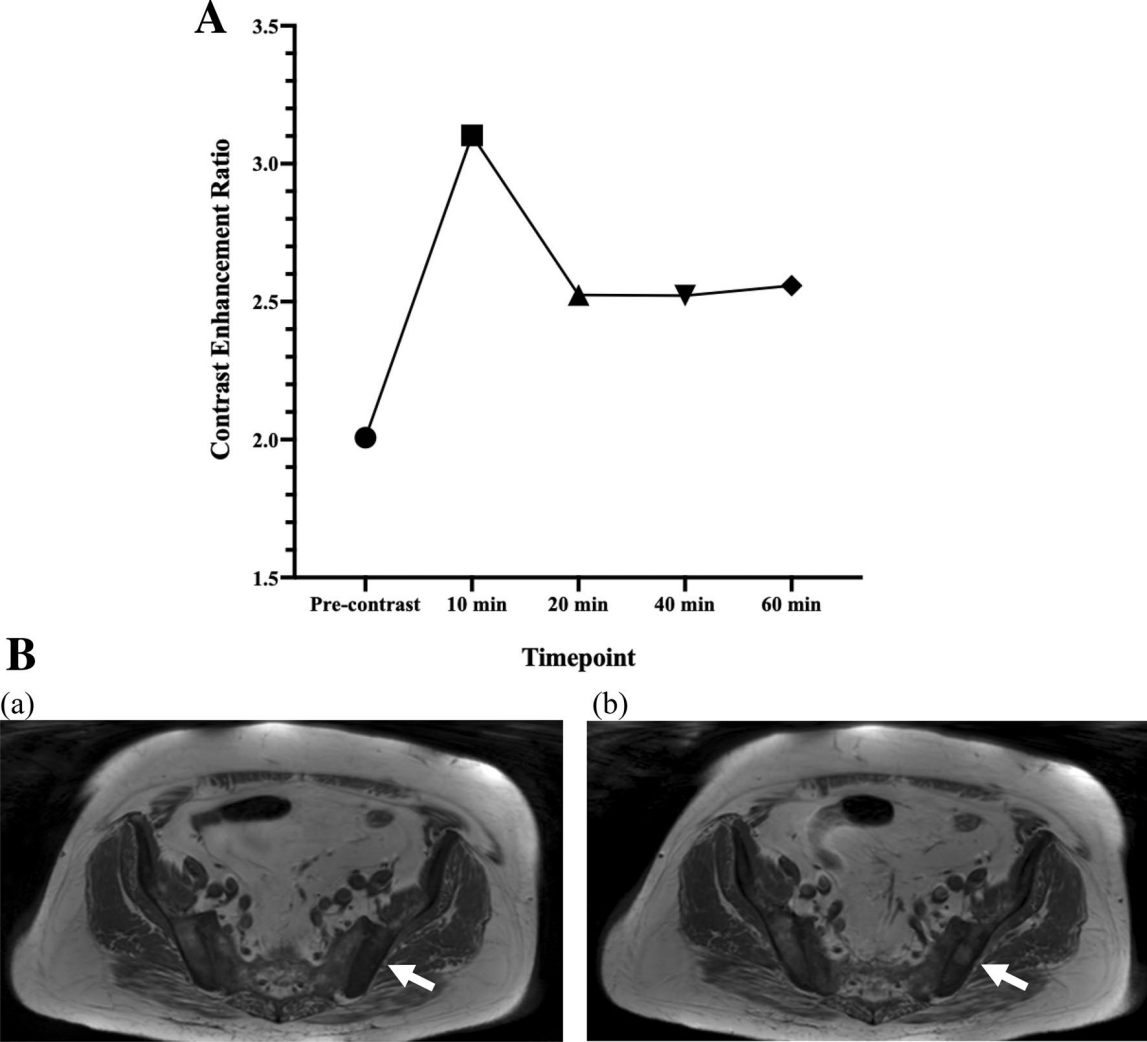
(**A**) Metastatic CRPC gadoxetate disodium enhancement pattern. Gadoxetate disodium enhancement in mCRPC peaks at 10 min post-injection, tapers at 20 min and retains enhancement over 60 min. (plotted as mean CER at each timepoint). (**B)** Pre and post gadoxetate disodium T1W MRI of iliac bone metastases. Baseline pre-contrast T1W MRI in patient with extensive bony metastases to the pelvis (a) and 60-minute post-gadoxetate disodium injection T1W MRI enhancing left iliac bony lesion (b). (see supplementary figure 1).

Twenty subjects completed gadoxetate disodium-enhanced MRI, 1 patient in the localized disease arm was excluded from analysis due to poor image quality secondary to biopsy related residual hemorrhage in the prostate. Enhancement of target lesion was seen in 17 of 19 (89.5%) patients. Consistent with prior studies, gadoxetate enhancement peaked at 10 min following injection^19^. Subgroup analysis was conducted to evaluate if (i) mCRPC tumors and (ii) localized prostate cancers take up and retain gadoxetate (Tables 1 and 2). In the mCRPC subgroup (n = 9), all subjects demonstrated gadoxetate enhancement. 77.8% (7/9) peaked at the 10-min time point and 2/9 (22.2%) peaked at the 60-min time point (Fig. 1A). The mCRPC subgroup demonstrated significant enhancement compared to pre-contrast images at 20-, 40- and 60-min timepoints (Table 1), indicating mCRPC takes up gadoxetate and retains it over time. An example of a gadoxetate enhancing lesion on T1W MRI is displayed in Fig. 1B. In the localized prostate cancer subgroup (n = 10), 80% (8/10) displayed gadoxetate enhancement, the 10-min time point was the only timepoint where enhancement was significantly elevated compared to pre-contrast images (*p* = 0.013). At no other later timepoints did the target lesion enhance significantly (Table 2), indicating localized prostate cancer initially enhances with gadoxetate but does not retain it over time. Gadoxetate enhancement did not change with lesion Gleason scores or baseline PSA (data not shown).

**Table 1:** **Gadoxetate disodium enhancement over time in metastatic CRPC subgroup.** Metastatic CRPC takes up gadoxetate disodium and demonstrated delayed enhancement.

Timepoints	Pre-contrast	Mean CER (SD)	*p* value**
10-min (n = 9)	2.01 ± 1.16	3.10 ± 2.61	0.055
20-min (n = 9)	2.01 ± 1.16	2.52 ± 1.27	0.004*
40-min (n = 9)	2.01 ± 1.16	2.52 ± 1.39	0.008*
60-min (n = 9)	2.01 ± 1.16	2.56 ± 1.45	0.004*
Average CER (n = 9)	2.01 ± 1.16	2.68 ± 1.58	0.004*

*Significant *p* value ** Statistical test used—Wilcoxon test.

**Table 2:** **Gadoxetate disodium enhancement over time in localized prostate cancer subgroup.** Localized prostate cancer takes up Eovist at 10-min timepoint but does not retain it over time.

Timepoints	Pre-contrast	Mean CER (SD)	*P* value**
10-min (n = 9)	1.86 ± 0.41	2.40 ± 0.34	0.013*
20-min (n = 10)	1.86 ± 0.41	2.21 ± 0.36	0.084
40-min (n = 9)	1.86 ± 0.41	2.06 ± 0.34	0.250
60-min (n = 10)	1.86 ± 0.41	2.06 ± 0.34	0.160
Average CER (n = 10)	1.86 ± 0.41	2.17 ± 0.33	0.084

* Significant *p* value ** Statistical test used—Wilcoxon test.

### OATP1B3 expression on IHC

**Figure 2:**
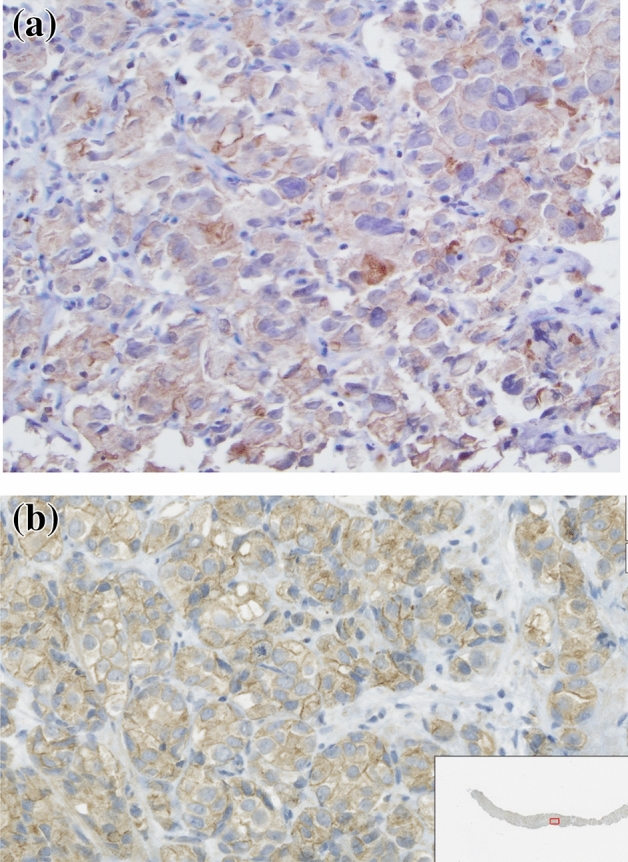
Immunohistochemistry anti-OATP1B3 antibody staining. (**a**) IHC slide of metastatic prostate cancer in lymph node. IHC using rabbit polyclonal anti-OATP1B3 antibody staining moderately positive (2+) with membranous and cytoplasmic distribution. (**b**) Slide of core biopsy of localized prostate cancer. IHC using rabbit polyclonal anti-OATP1B3 antibody staining moderately positive (2+) with membranous and cytoplasmic distribution.

The expression of OATP1B3 has previously been shown to have a strong correlation with gadoxetate uptake in hepatic cancers^17,21^. We hypothesized that prostate tumors expressing OATP1B3, as demonstrated on IHC, would have increased uptake. Fifteen subjects had tissue available for IHC, 9 localized and 6 mCRPC cases, 2 of which did not complete the gadoxetate disodium-enhanced MRI. Overall 12/15 (80%) stained positively and 3/15 (20%) negatively. 80% of local prostate cancer cases and 66.67% of metastatic CRPC cases stained positive for OATP1B3. The staining pattern demonstrated membranous staining of tumor cells with cytoplasmic blushing (Fig. 2). Similar to previous studies, we found OATP1B3 expression on IHC trended towards higher CER between 20–40 min (*p* ≤ 0.064) and was associated with gadoxetic enhancement at 60 min, mean CER of 2.02 vs. 1.47 (*p* = 0.042) (Table 3).

**Table 3:** **Gadoxetate disodium enhancement based on OATP1B3 Expression on IHC.** Total of 15 patients, 9 localized and 6 mCRPC. OAPT1B3 expression was associated with increased retention of gadoxetate disodium at 60-min timepoint.

Timepoints	2nd IHC OATP1B3 + (n = 12)	2nd IHC OATP1B3- (n = 3)	P-value **
10-min (n = 13)	2.33 ± 0.33	1.6 ± 0.65	0.204
20-min (n = 13)	2.20 ± 0.34	1.52 ± 0.47	0.062
40-min (n = 12)	2.01 ± 31	1.41 ± 0.42	0.064
60-min (n = 13)	2.02 ± 0.32	1.47 ± 0.41	0.042*

*Significant *p* value ** Statistical test used—Mann Whitney Test.

### Genotype

We completed a comprehensive assessment of fifty-one SNPs in the three genes associated with gadoxetate transport; *SLC10A1* (7), *SLCO1B3* (8) and *SLCO1B1* (36) (Supplementary Table 2). The *SLCO1B3* 334T>G and 699G>A polymorphisms have been associated with OATP1B3 transporter activity and demonstrated to be predictive and prognostic markers in men with prostate cancer^6,7,9,10^. We hypothesized that OATP1B3 variants associated with increased androgen uptake and poor prostate cancer outcomes would enhance and retain gadoxetate more efficiently. Twenty-one patient samples were available for genotyping: 4 African Americans, 1 Asian, and 16 Caucasians. Consistent with previous genotype frequencies in Caucasians and African Americans^23^, our genotyping results showed 4 (19%) patients were wild-type and 17 (81%) patients were homozygous variant;18 (85.7%) patients had corresponding MRI data for analysis. No significant difference in CER was seen based on variant *SLCO1B3* genotypes at any timepoint (Supplementary Table 3*).* Eighteen patient samples were available to be genotyped by Pharmacoscan and 16 had corresponding MRI data. Upon evaluation of 36 polymorphisms in *SLCO1B1*, 521T>C V174A (rs4149056) SNP (*n* = 2) demonstrated a putative association with gadoxetate enhancement at 40- and 60- minute timepoints (*p* ≤ 0.038) (Supplementary Table 4). Of these patients, which demonstrated the second and third highest enhancements in our cohort, one patient carried the N130D-V174A haplotype and the second patient, with a non-informative genotype, may have carried this haplotype. However, genotype-predicted *SLCO1B1* transporter phenotype (i.e., poor-, intermediate-, or normal-transporter status) was not associated with enhancement (Supplementary Table 5 & Supplementary Fig. 2). No association was seen between gadoxetate enhancement and *SLC10A1* or the six further *SLCO1B3* variants evaluated (*p* > 0.05).

## Discussion

Our pilot study demonstrates that prostate tumors enhance and retain gadoxetate disodium on T1W MRI, thus exploiting de novo OATP1B3 expression in prostate tumors for *in-vivo* imaging purposes. This enhancement pattern was only evident in mCRPC, which demonstrated delayed enhancement at 20-, 40- and 60-min timepoints, unlike conventional gadolinium-based contrast agents (GBCAs). This is consistent with both preclinical cell line studies which showed OATP1B3 was the second most differentially expressed transporter between androgen-sensitive and CRPC cell lines, and tumor tissue studies which showed almost fourfold higher OATP1B3 expression between primary prostate and mCRPC tumors^5,7^. In contrast, the primary prostate cancer cases enhanced at the 10-min timepoint, consistent with prior studies demonstrating gadoxetate enhances all vascular structures and organs due to its low molecular weight properties^19^, and did not demonstrate any retention of contrast over subsequent timepoints, similar to conventional GBCAs.

OATP1B3 expression was previously associated with a higher gadoxetate uptake in hepatic cancers^17,21^, and in the present study we demonstrate that OATP1B3 expression on IHC trended towards higher CER between 20 and 40 min and was associated with gadoxetate enhancement at 60 min. These data suggest de novo OATP1B3 expression is also associated with increased uptake kinetics, particularly in metastatic prostate cancers. OATP1B3 has more than one isoform, liver-type (LT) and cancer-type (CT), and the CT-isoform is localized mainly in the cytoplasm with modest transport activity^24^. Current commercially available antibodies cannot distinguish between OATP1B3 isoforms and we were unable to determine which isoform was responsible for gadoxetate uptake due to limited sampling constraints in this pilot study. OATP1B3 isoforms have the potential to significantly impact gadoxetate enhancement, given their location and transporter function differences. Future directions of study should include characterization of (i) the expression of OATP1B3 isoforms in mCRPC tissues, (ii) the function of CT-OATP1B3 in prostate cancer, and (iii) tumoral androgen uptake kinetics in those cancers with high gadoxetate disodium enhancement.

Gadoxetate disodium is a substrate for OATP1B3, OATP1B1, and NTCP^16,17^. *SLCO1B3* 334T>G and 699 G>A polymorphisms have been shown to affect androgen transport kinetics and are associated with clinical outcomes in prostate cancer^5–7^. SNPs in *SLCO1B3* have been shown to impact gadoxetate transport kinetics in *in-vitro* and *in-vivo* liver cells^19,20^. No difference in gadoxetate enhancement was associated with *SLCO1B3* genotypes in our study. Two commonly occurring variants of *SLCO1B1*, 388A>G and 521T>C, are associated with markedly reduced hepatic uptake of multiple drugs^20^. *In-vitro* cell studies demonstrated that co-transfection of *SLCO1B1* 388A>G and 521T>C (SLCO1B1*15), but not *SLCO1B1* 521T>C alone (SLCO1B1*5), resulted in significantly decreased uptake of gadoxetate^19^. The importance of the 338G-521C haplotype was observed in this study as well, of the three patients with the highest enhancements at 40 and 60 min, one individual harbored the 338G-521C haplotype, and one individual harbored a non-informative genotype (i.e. was heterozygous at both sites) and so could have also harbored this haplotype. The counter-intuitively greater gadoxetate enhancement in prostate cancer cells with this haplotype indicates this effect may be caused by slower hepatic clearance of gadoxetate in patients harboring this haplotype rather than differential uptake in mCRPC itself. This view is also supported by studies demonstrating mCRPC has a rather low expression of OATP1B1 in comparison with other uptake transporters^7^.

OATP1B3 also transports anticancer drugs such as paclitaxel and docetaxel^3^ and preclinical studies have demonstrated that loss of OATP1B3 results in taxane resistance^12^. Current imaging modalities cannot predict treatment failure or resistance. Gadoxetate disodium-enhanced MRI may have the potential to be a noninvasive predictive biomarker in the assessment of which patients would benefit from anticancer drugs transported by OATP1B3, for example docetaxel and cabazitaxel in prostate cancer. Since OATP1B3 also transports androgens^5^, its gadoxetate-predicted function may also have prognostic significance in prostate cancers and/or predict which patients are likely to more-efficiently import androgens and therefore progress more-rapidly on therapies that restrict androgen function or production. Future prospective studies should address if gadoxetate enhancement has a prognostic role in prostate cancer outcomes and/or a predictive role in treatment response and/or stratification.

Limitations of our study include small sample size, assessment of total OATP1B3 expression in our IHC analysis, and our inability to distinguish between LT- and CT-OATP1B3 expression.

## Conclusion

We present the first study to document that mCRPC lesions demonstrate enhancement after injection of gadoxetate disodium on T1W MRI and retain the contrast over 60 min. Gadoxetate disodium-enhanced MRI warrants further study as a potential non-invasive prognostic and therapeutic biomarker in prostate cancer.

## Supplementary Information

Supplementary information.
