# Supplementary material for: Pilot study of gadoxetate disodium-enhanced mri for localized and metastatic prostate cancers
Source: Sci Rep. 2021 Mar 11;11:5662. doi: 10.1038/s41598-021-84960-w (PMC7952731; doi:10.1038/s41598-021-84960-w)

**Manuscript Title:** Pilot Study of Gadoxetate Disodium-Enhanced MRI for Localized and Metastatic Prostate Cancers”

**Authors:**

Sarah E. Lochrin^1^*, Baris Turkbey^2^*, Billel Gasmi^3^, Keith Schmidt^1^, Jonathan D. Strope^1^, Cindy H. Chau^4^, Tristan M. Sissung^1^, Douglas K. Price^4^, Lisa Cordes^1^, Suzana Markolovic^1^, Bradford J. Wood^5^, Peter A. Pinto^6^, Yolanda L. McKinney^2^, Joanna H. Shih^7^, Elliot Levy^8^, Ravi Madan^4^, William Dahut^4^, Peter L. Choyke^2^, Maria Merino^3^, William D. Figg^1,4^.

*co-first authors

*From the*: ^1^Clinical Pharmacology Program, ^2^Molecular Imaging Branch, ^3^Translational Surgical Pathology Section, ^4^Genitourinary Malignancies Branch, ^5^Center for Interventional Oncology, ^6^Urologic Oncology Branch, ^7^Biometric Research Program, ^8^Radiology and Imaging Sciences, Center for Cancer Research, National Cancer Institute, Bethesda, MD.

*Corresponding author*:

William D. Figg, PharmD, Genitourinary Malignancies Branch, National Cancer Institute, 9000 Rockville Pike, Building 10, Room 5A03, Bethesda, MD 20892; Ph. (240) 760-6179, Fax. (240) 541-4536. Email: [figgw@mail.nih.gov](mailto:figgw@mail.nih.gov)

**Supplementary Data**

**Supplementary Table 1.**

Patient Characteristics


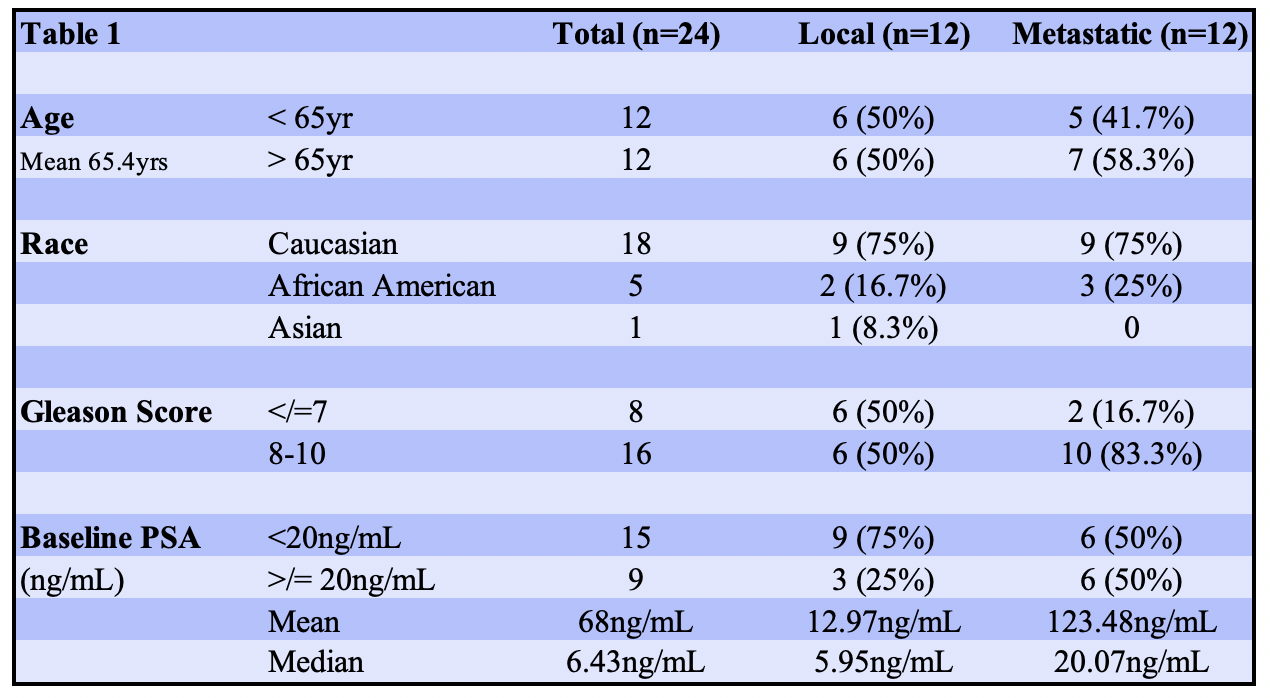


**Supplementary Table 2**

Genes and polymorphisms assessed via Pharmacoscan.

| ***SLC10A1*** | rs4646285 | ***SLCO1B1*** | rs4149015 | ***SLCO1B3*** | rs4149143 |
| --- | --- | --- | --- | --- | --- |
| (NTCP) | rs55645214 |  | rs72559747 |  | rs2053098 |
|  | rs61745930 |  | rs59502379 |  | rs11045585 |
|  | rs2296651 |  | * rs4363657 |  | rs3764006 |
|  | rs72547507 |  | # rs11045872 |  | rs4149117 |
|  | rs72547506 |  | * rs4149081 |  | rs4149118 |
|  | rs56903885 |  | * rs11045879 |  | rs7311358 |
|  |  |  | rs34671512 |  | rs7977213 |
|  |  |  | rs55737008 |  |  |
|  |  |  | rs2291073 |  |  |
|  |  |  | rs56061388 |  |  |
|  |  |  | rs4149036 |  |  |
|  |  |  | rs2306283 |  |  |
|  |  |  | # rs11045818 |  |  |
|  |  |  | # rs11045819 |  |  |
|  |  |  | rs72559745 |  |  |
|  |  |  | rs77271279 |  |  |
|  |  |  | * rs4149056 |  |  |
|  |  |  | * rs4149057 |  |  |
|  |  |  | rs2291075 |  |  |
|  |  |  | # rs11045821 |  |  |
|  |  |  | rs11045852 |  |  |
|  |  |  | rs11045853 |  |  |
|  |  |  | rs4149032 |  |  |
|  |  |  | rs2306282 |  |  |
|  |  |  | rs72559746 |  |  |
|  |  |  | rs56101265 |  |  |
|  |  |  | rs373327528 |  |  |
|  |  |  | rs142965323 |  |  |
|  |  |  | rs59113707 |  |  |
|  |  |  | rs140790673 |  |  |
|  |  |  | rs79135870 |  |  |
|  |  |  | rs139257324 |  |  |
|  |  |  | rs55901008 |  |  |
|  |  |  | rs56387224 |  |  |
|  |  |  | rs72559748 |  |  |

***/#** rsIDs linked in our cohort

**Supplementary Table 3.**

Gadoxetate disodium enhancement based on OATP1B3 S112A & M233I Genotype.

| n=21 | Wild Type  (n=3) | Variant  (n=15) | P-value * |
| --- | --- | --- | --- |
| 10-minutes  (n=17) | 2.29  +/-0.30 | 2.97  +/- 2.03 | 0.6590 |
| 20-minutes  (n=18) | 2.05  +/- 0.43 | 2.50  +/- 0.94 | 0.5145 |
| 40-minutes  (n=17) | 1.95  +/-0.51 | 2.45  +/-1.06 | 0.6765 |
| 60-minutes  (n=18) | 1.9  +/-0.52 | 2.45  +/-1.08 | 0.4410 |

* Statistical test used - Mann Whitney test. **Significant p value. Values: Mean CER (SD).

Legend: SLCO1B3 genotype did not impact gadoxetate disodium enhancement at any timepoint.

**Supplementary Table 4**.

Gadoxetate disodium enhancement based on OATP1B1 521T>C (rs4149056) V174A Genotype.

| n=16 | Wild Type (TT)  (n=14) | Heterozygote (T/C)  (n=2) | P-value * |
| --- | --- | --- | --- |
| 10-minutes  (n=17) | 2.80  +/- 2.10 | 3.65  +/- 0.07 | 0.0762 |
| 20-minutes  (n=18) | 2.27  +/- 0.74 | 3.78  +/- 1.38 | 0.0667 |
| 40-minutes  (n=17) | 2.13  +/- 0.76 | 4.23  +/- 0.89 | 0.0381** |
| 60-minutes  (n=18) | 2.17  +/- 0.82 | 4.02  +/- 1.58 | 0.0333** |

*Mann Whitney test. **Significant p value. Values: Mean CER +/- SD.

**Supplementary Table 5.**

*SLCO1B1* haplotypes and corresponding genotype-predicted *SLCO1B1* transporter phenotype.

| n=18 | ***SLCO1B1* Haplotype** | **Transporter Phenotype** | **Number of patients** |
| --- | --- | --- | --- |
|  | *1A/*1A | normal | 8 (44.44%) |
|  | *1B/*1B | normal | 1 (5.56%) |
|  | *1A/*35,*1B/*22 | normal/increased, normal/unknown | 2 (11.11%) |
|  | *1B/*35 | normal/increased | 1 (5.56%) |
|  | *1A/*14,*1B/*4 | normal/increased, normal/unknown | 4 (22.22%) |
|  | *1A/*15,*1B/*5 | normal/decreased | 1 (5.56%) |
|  | *1B/*15 | normal/decreased | 1 (5.56%) |

**Supplementary Figure 1.**

Preliminary IHC analysis was conducted using goat polyclonal anti-OATP1B3 antibody (Santa Cruz Biotech, dilution 1:50 in PBST), however staining of all specimens could not be completed as this antibody was discontinued during trial enrollment. Displayed here is an image of a metastatic prostate cancer deposit in the bone marrow which stained strongly positive (3+) from the left ilium corresponding with the lesion seen enhancing on gadoxetate disodium MRI at the 60-minute timepoint displayed in Figure 2A.

**
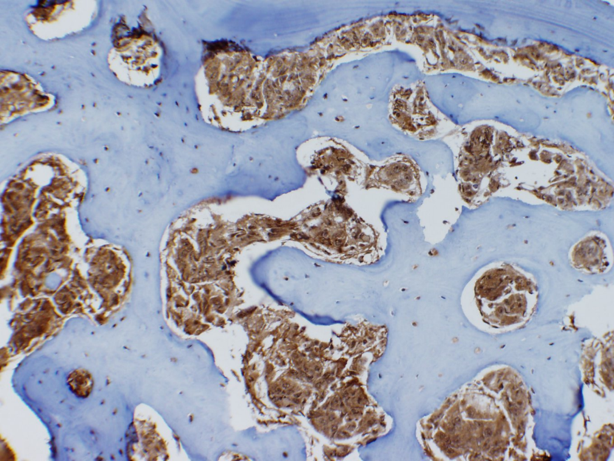

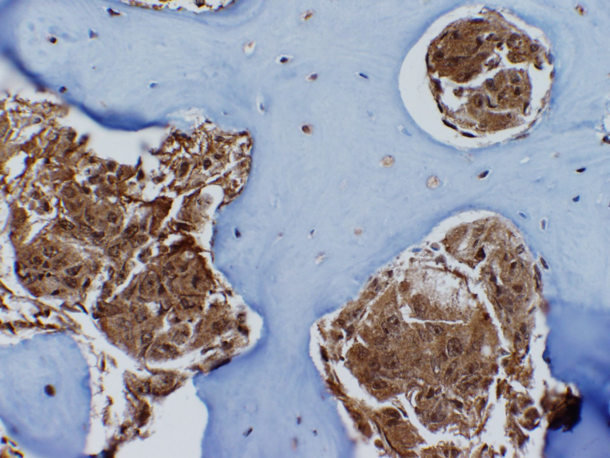
**

**Supplementary Figure 2.**

Gadoxetate Disodium enhancement in seven *SLCO1B1* haplotypes at each timepoint. No significant difference seen.


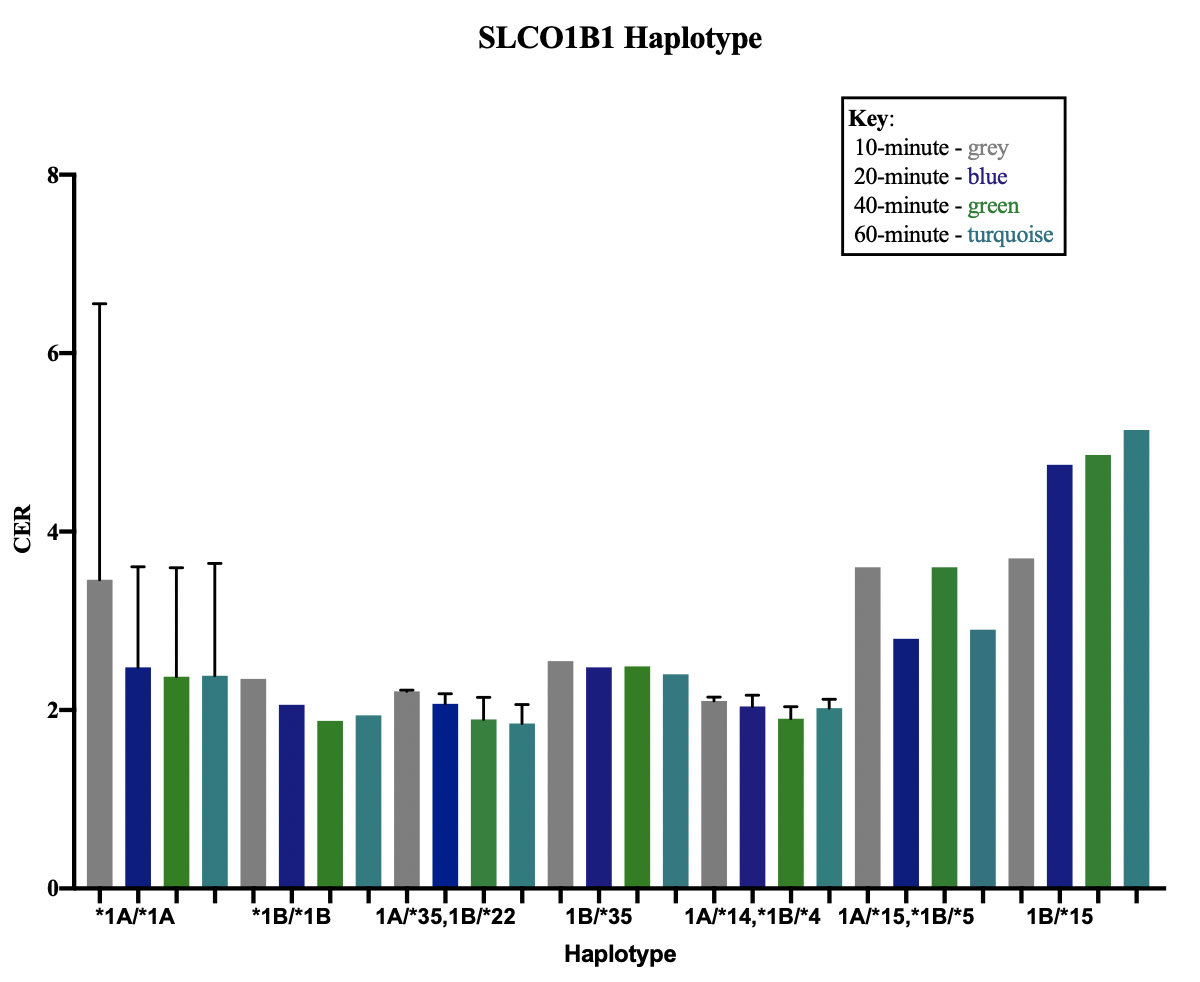

Supplement: Supplementary file 1 — Supplementary information. [file 41598_2021_84960_MOESM1_ESM.docx]
